# Chitosan Micro-Membranes with Integrated Gold Nanoparticles as an LSPR-Based Sensing Platform

**DOI:** 10.3390/bios12110951

**Published:** 2022-11-01

**Authors:** Diana I. Meira, Manuela Proença, Rita Rebelo, Ana I. Barbosa, Marco S. Rodrigues, Joel Borges, Filipe Vaz, Rui L. Reis, Vitor M. Correlo

**Affiliations:** 1 Physics Center of Minho and Porto Universities (CF-UM-UP), Campus de Azurém, University of Minho, 4800-058 Guimarães, Portugal; 2 3 B’s Research Group, I3Bs—Research Institute on Biomaterials, Headquarters of the European Institute of Excellence on Tissue Engineering and Regenerative Medicine, Biodegradables and Biomimetics of University of Minho, AvePark—Parque de Ciência e Tecnologia, Zona Industrial da Gandra, 4805-017 Guimarães, Portugal; 3 ICVS/3B’s—PT Government Associated Laboratory, AvePark, Zona Industrial da Gandra S. Claudio do Barco, Caldas das Taipas, 4806-909 Guimarães, Portugal; 4 LaPMET—Laboratory of Physics for Materials and Emergent Technologies, University of Minho, Campus de Gualtar, 4710-057 Braga, Portugal

**Keywords:** plasmonics, Au nanoparticles, chitosan membrane, high-resolution LSPR spectroscopy, optical sensing

## Abstract

Currently, there is an increasing need to develop highly sensitive plasmonic sensors able to provide good biocompatibility, flexibility, and optical stability to detect low levels of analytes in biological media. In this study, gold nanoparticles (Au NPs) were dispersed into chitosan membranes by spin coating. It has been demonstrated that these membranes are particularly stable and can be successfully employed as versatile plasmonic platforms for molecular sensing. The optical response of the chitosan/Au NPs interfaces and their capability to sense the medium’s refractive index (RI) changes, either in a liquid or gas media, were investigated by high-resolution localized surface plasmon resonance (HR-LSPR) spectroscopy, as a proof of concept for biosensing applications. The results revealed that the lowest polymer concentration (chitosan (0.5%)/Au-NPs membrane) presented the most suitable plasmonic response. An LSPR band redshift was observed as the RI of the surrounding media was incremented, resulting in a sensitivity value of 28 ± 1 nm/RIU. Furthermore, the plasmonic membrane showed an outstanding performance when tested in gaseous atmospheres, being capable of distinguishing inert gases with only a 10^−5^ RI unit difference. The potential of chitosan/Au-NPs membranes was confirmed for application in LSPR-based sensing applications, despite the fact that further materials optimization should be performed to enhance sensitivity.

## 1. Introduction

Localized surface plasmon resonance (LSPR) sensing is a signal detection technique that has been significantly explored in the field of biosensing. LSPR arises from the collective oscillation of electrons confined in metallic nanostructures (NSTs) when excited by incident light of a wavelength that is much larger than the size of the nanostructure, exhibiting a unique extinction (absorption and scattering) spectrum—the LSPR band [1]. Both the intensity and peak location of the LSPR band are highly dependent on the NSTs’ shape, size, and composition, as well as the refractive index (RI) of the surrounding medium [2,3]. Changes in the gas or liquid nano-environment and analyte binding to the plasmonic NSTs cause a resonance peak shift due to a refractive index change, which can be used and explored for label-free and real-time detection of gases [4], liquids [5], and (bio)molecules [6,7]. LSPR platforms offer label-free, sensitive, rapid, robust, and versatile analyte detection, being, therefore, a desirable detection technology for a variety of biosensing applications such as biomedical [8], environmental [9], agricultural [10], and food industry [11] applications, among others.

LSPR biosensors rely on a diversity of metallic NSTs, noble metal nanoparticles (NPs), preferred due to the strong scattering of light in the visible range. Furthermore, due to their outstanding optical properties and high stability, gold (Au) NPs have become widely used in LSPR biosensors.

In most LSPR biosensors, NPs are embedded in a variety of substrates that control their equidistance, their 2D or 3D organization, the analyte diffusion, and biosensor stability [1,12,13]. These parameters also significantly affect the performance of the biosensor. Nanocomposite thin films are attractive for LSPR since they allow (i) a homogeneous distribution of metallic NSTs in the form of metallic NPs, (ii) porous media for analyte diffusion, (iii) NPs 3D dispersion, and (iv) lower thickness for optimal optical detection with in-house built LSPR systems [14,15,16]. Several “host” matrices have been explored and reported for LSPR sensing, including metallic oxides and polymeric thin films. Polymers are low-cost materials that can be deposited in several substrates with diverse shapes and sizes. Additionally, the use of porous polymer matrices may allow permeability, stability, accumulation of analyte ions, and increased sensitivity [17]. Moreover, due to the increasing need to develop sensing platforms able to adapt to non-planar surfaces, polymers are being used to create flexible substrates, which allow a rapid, real-time, and cost-effective monitoring and a rapid and high processability, which can lead to a fast scale up. Thus, the combination of polymers with metallic NPs, showing promising sensing properties, has been explored [18], through several manufacturing techniques, with the most common being: spin-coating [19,20], lithography [17,21], and layer-by-layer deposition [22,23].

Chitosan is a biodegradable and biocompatible natural polymer that has been widely used in different forms (e.g., gels, films, particles, membranes, or scaffolds) in a large number of applications, ranging from biomedical to industrial areas. It is a cationic polysaccharide produced by the alkaline deacetylation of chitin, a polysaccharide that can be found in shells of marine crustaceans and cell walls of fungi [24,25]. Chitosan, when integrated with Au NPs, can be an excellent choice for LSPR sensing platforms since it presents several advantages: natural-based, transparency, easily accessible and scaled up, cheap, flexible, and potential for wearable and point-of-care applications, among others.

Despite LSPR being a well-explored and sensitive technique, it faces some limitations. Sensitivity, detection limits, and utility in point-of-care diagnostic devices are key parameters that should be optimized for LSPR biosensors to achieve their full potential. Additionally, LSPR-based sensing could be extremely challenging, for instance, for gas detection, since the refractive index (RI) changes can be extremely small.

This research article aims to present a proof of concept of the nanoplasmonic sensing application of Au NPs embedded in chitosan membranes using a high-resolution LSPR (HR-LSPR) spectroscopy system. The system is a combination of a modular spectrometer and software developed by the group [26], which allows the processing of data to identify shifts in the T-LSPR wavelength peak position with a resolution as low as 0.005 nm.

The nanoplasmonic membrane production is described, as well as the characterization and evaluation of its final sensing performance.

## 2. Materials and Methods

### 2.1. Materials

All chemicals, including chitosan medium molecular weight (85% deacetylation), were purchased from Laborspirit (Loures, Portugal) unless stated otherwise.

Ethanol (70%) was purchased from AGA- Álcool e Géneros Alimentares S.A. (Loures, Portugal).

Au NPs (20 nm diameter, OD 1, stabilized suspension in 0.1 mM PBS, reactant free) were purchased from Merck (Darmstadt, Germany).

### 2.2. Methods

#### 2.2.1. Chitosan Purification

Before membrane preparation, commercial medium molecular weight chitosan was purified to remove impurities. In summary, chitosan (1 wt.%) was dissolved in acetic acid (2 wt.%) and left stirring overnight. Afterward, the solution was filtered and washed with distilled water several times. The pH of the solution was adjusted to 8 by stirring the solution and adding slowly 2M NaOH to promote the precipitation of the polymer. The precipitated chitosan was washed with distilled water until it reached pH 7, filtered, and rewashed with several ethanol–water solutions. In the end, the chitosan was frozen (−80 °C) and freeze-dryer.

#### 2.2.2. Chitosan/Au-NPs Membrane Production

**Figure 1:**
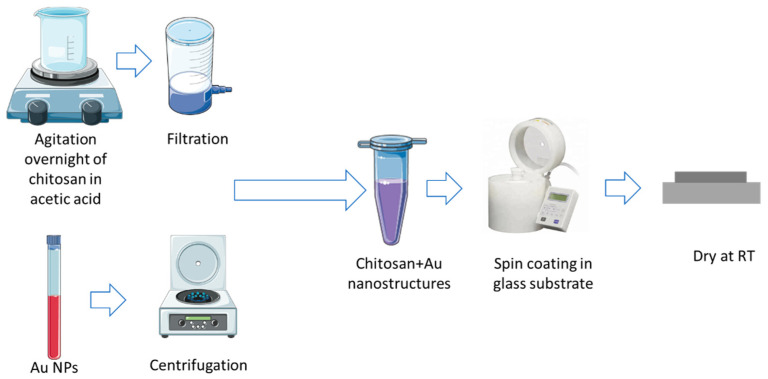
Schematic representation of methodology for chitosan/Au-NPs membrane production.

Chitosan solutions with four distinct polymer concentrations (0.5, 0.75, 1, and 1.5% (*w/v*)) were prepared in an acetic acid (1.5 wt.%) solution and left stirring overnight. Afterward, the solutions were filtered to remove possible impurities. Meanwhile, the Au NPs solution was centrifuged at 13,500 rps for 30 min at room temperature. After centrifugation, the supernatant was removed, and 5 µL of the Au NPs were added to 15 µL of the chitosan solution. In the final step, the 20 µL solution was dropped in a previously plasma-treated (200 w, 90 s) glass, spin-coated (150 rpm, 50 rpm/s, 1 min), and left to dry in the hood. The schematic production of the chitosan/Au-NPs membranes is exhibited in Figure 1.

#### 2.2.3. Surface Characterization Using Atomic Force Microscopy (AFM)

The distribution and morphology of the chitosan/Au-NPs membranes were evaluated by atomic force microscopy (AFM) using AFM Dimension Icon (from Bruker, Billerica, MA, USA) equipment, operating in PeakForce Tapping (ScanAsyst) in air. AFM cantilevers (ScanAsyst-Air, Bruker) made of silicon nitride with a spring constant of 0.4 N/m and frequency of 70 kHz were used. The three-dimensional (3D) AFM topography data were analyzed with the freely available Gwyddion software (version 2.50).

#### 2.2.4. Sensitivity Tests Using HR-LSPR Spectroscopy

**Figure 2:**
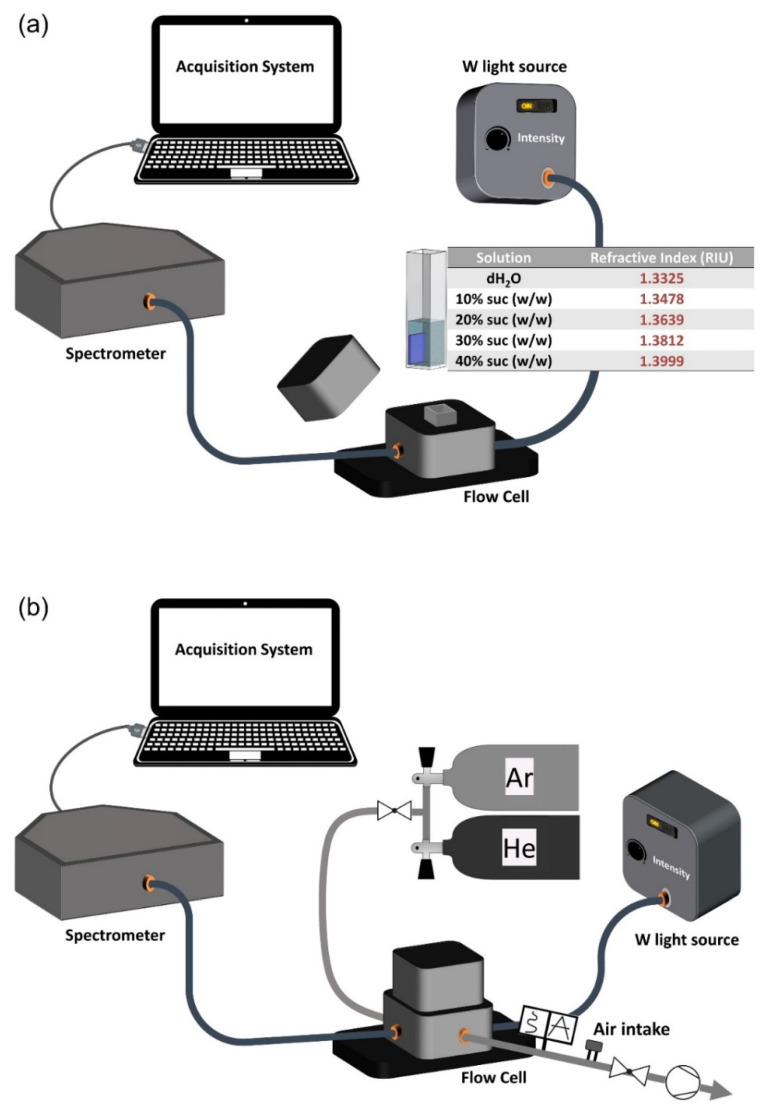
Schematic representation of experimental setup for HR-LSPR sensing, adapted to the liquid (**a**) and gas (**b**) experimental procedure.

The chitosan/Au-NPs membranes are being developed to be used as nanoplasmonic sensing devices. Thus, it is important to demonstrate their sensitivity, through the refractive index sensitivity (RIS) and the limit of detection (LOD) values determination, by measuring LSPR band shifts and establishing the signal-to-ratio resulting from the medium’s refractive index change. Figure 2 presents the schematics of the sensing setup connected to the HR-LSPR spectroscopy system, previously described in [4], where the transmittance spectra of the most promising nanoplasmonic membrane were measured—the transmittance minimum (T-LSPR peak) corresponds to the maximum of light extinction.

- Liquid Sensitivity Tests

Regarding the liquid sensitivity tests, the most promising nanoplasmonic membrane sample was exposed to several solutions with different refractive indices (water and crescent concentrations (wt./wt.) of sucrose solutions) to evaluate its sensitivity. The sensing setup equipment was adapted to the liquid experimental procedure, as Figure 2a illustrates. The sensing testing was based on immersion of the representative sample in each solution for 1 min, and each transmittance spectrum was acquired using an integration time of 4 ms and an average of 500 scans. Afterward, membranes were subjected to a cleaning step (water and ethanol) before immersion into the following solution. The procedure was repeated for 5 cycles for each solution. Finally, the NANOPTICS software (version 2022a) developed by Marco S. Rodrigues from University of Minho (Guimarães, Portugal) processed data to identify changes in the T-LSPR wavelength peak position [26]. RIS was calculated using the equation RIS = Δλ/Δη (nm/RIU), assuming a semi-infinite layer of the aimed solution, where Δλ represents the wavelength peak shift and Δη the difference of the refractive index of the surrounding media [27].

- Gas Sensitivity Tests

In addition to liquid sensitivity tests, the gas sensing potential of the same chitosan/Au-NPs membrane was evaluated by varying the refractive index of the medium surrounding the nanoplasmonic membrane with pure gases of He and Ar (RI (He) = 1.000009 and RI (Ar) = 1.00007025, at 2.6 × 10^4^ Pa at room temperature). For that, the sensing setup equipment was adapted with a vacuum system, as illustrated in Figure 2b, which kept the flow cell with the nanoplasmonic membrane under a primary vacuum at 1.8 × 10^2^ Pa. The sensing procedure was initiated when the gaseous atmosphere inside the flow cell was switched between pure He and pure Ar, every 120 s, while the T-LSPR band was monitored in real time. The partial pressure of each gas was maintained at 2.6 × 10^4^ Pa at room temperature. The reproducibility of the sensing procedure was confirmed by exposing the chitosan/Au-NPs membrane to several He/Ar cycles. The peak position of the LSPR band (wavelength and transmittance coordinates) over time, the average shifts caused by the exposure to different gaseous atmospheres, and the signal-to-noise ratio (SNR) of the measurements were calculated using the NANOPTICS software.

## 3. Results and Discussion

### 3.1. Optical (T-LSPR) Response of Chitosan/Au-NPs Membranes

**Figure 3:**
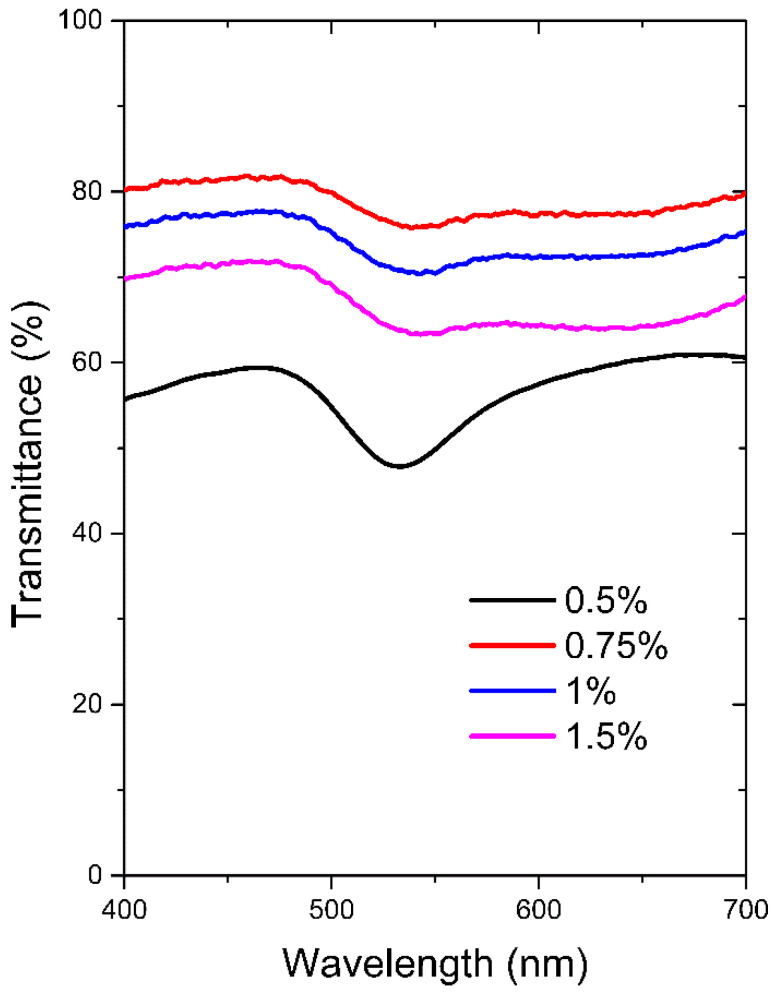
T-LSPR spectra of produced chitosan membranes with Au NPs with different polymer concentrations (0.5, 0.75, 1, and 1.5%).

In order to access the sensing ability of the plasmonic chitosan/Au-NPs membranes, the optical response of the samples was firstly evaluated using representative samples of different concentrations. Therefore, the influence of the polymeric concentration (0.5, 0.75, 1, and 1.5%) on the optical (T-LSPR) response of nanoplasmonic membranes was analyzed, as demonstrated in Figure 3.

In the transmittance spectra of chitosan/Au-NPs membranes, the specific LSPR band due to the plasmonic NPs is noticeable at about 540 nm (Figure 3) for all formulations, thus confirming its presence in the chitosan membranes. However, the membrane with the lowest chitosan concentration (0.5% (*w/v*)) presented a more intense and well-defined T-LSPR band, while the remaining conditions revealed a smoother T-LSPR band and an almost negligible plasmonic behavior. The higher chitosan concentrations probably hindered fine Au NPs dispersion into the membrane, thus diminishing the concentration of plasmonic metal and, hence, the LSPR absorption became very faint. On the other hand, the dispersion of Au NPs on the lowest chitosan concentration membrane was successfully achieved, promoting a pronounced T-LSPR signal.

Taking into account the optical response results, the chitosan (0.5%)/Au-NPs membrane was considered the most promising experimental condition to be further characterized and explored as an LSPR-based sensing transducer.

### 3.2. Surface Characterization of Chitosan (0.5%)/Au-NPs Membrane

**Figure 4:**
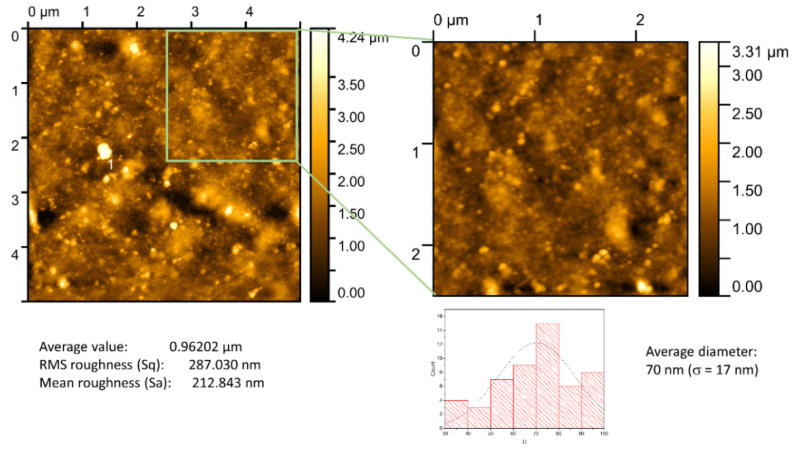
AFM surface topography of chitosan (0.5%)/Au-NPs membrane and its surface characteristics analysis, including the average height value, RMS roughness (Sq), mean roughness (Sa), and the average diameter of NPs.

Considering that the most promising sensing nanoplasmonic membrane was the chitosan (0.5%)/Au-NPs membrane, the evaluation of the surface characteristics becomes relevant to understanding its sensing ability. Therefore, the topographic analysis conducted by AFM is displayed in Figure 4, showing the surface nanotexture. On the surface of the membrane, it is possible to observe the Au NPs distributed along the chitosan matrix, with an average RMS roughness estimated at 287 nm. The membrane presents nanostructures with average sizes of around 70 nm (σ = 17 nm), which corroborates the T-LSPR measurement results [28]. Considering the initial size of the Au NPs in the colloidal solution (about 20 nm) the obtained nanostructures in the membrane with higher dimensions can be the result of agglomerates, promoted by the strong tendency of colloidal Au NPs to aggregate due to Van der Waals attraction [29]. In any event, the quantity of isolated NPs contributing to the LSPR behavior must be relevant, taking into account the optical transmittance spectrum.

### 3.3. T-LSPR Liquid Sensing of Chitosan (0.5%)/Au-NPs Membrane

**Figure 5:**
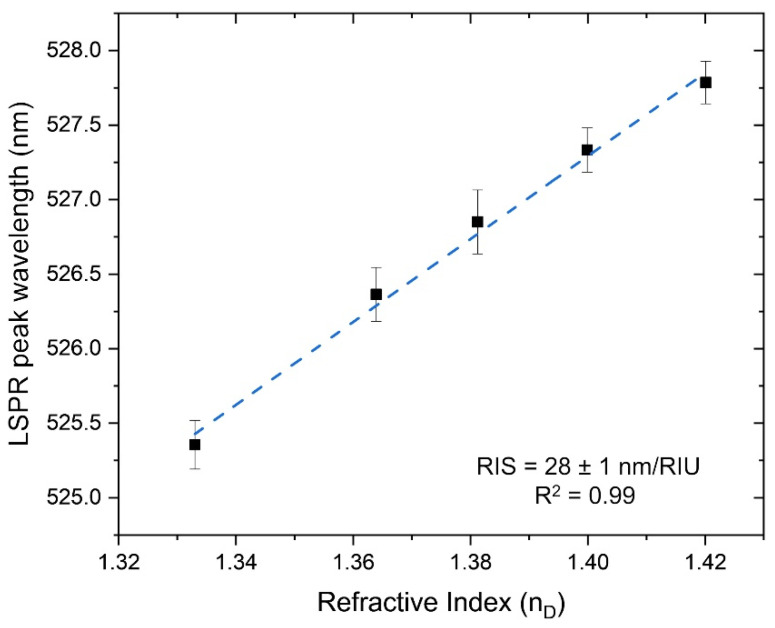
T-LSPR wavelength peak position of chitosan (0.5%)/Au-NPs plasmonic membrane as a function of the refractive index of the different solutions (water and sucrose at 20, 30, 40, and 50% (wt/wt)). The solid line is a linear fit applied to the data.

In this particular LSPR platform, the sensitivity test is based on the ability to differentiate refractive index variations in the surrounding media through a T-LSPR band peak shift. Therefore, the chitosan (0.5%)/Au-NPs nanoplasmonic membrane’s sensing ability was firstly evaluated through the performance of an RIS test using different sucrose solutions with several concentrations, thus increasing refractive index values, as exemplified in Figure 2a. In fact, the chitosan (0.5%)/Au-NPs representative sample changed its optical response due to the refractive index variations in its surrounding media, once the T-LSPR wavelength peak position suffered a consequent redshift as the test solutions revealed a higher refractive index, as demonstrated in Figure 5. This association led to the determination of the RIS value, being 28 ± 1 nm/RIU, with a correlation coefficient R^2^ of 0.99. According to the IUPAC definition [30], the LOD value was estimated in this case to be 1.339 RIU, corresponding to a 0.007 RIU shift from the water reference. The sensitivity of the nanoplasmonic membrane is similar to magnetron-sputtered nanocomposite thin film containing Au NPs dispersed on a titanium dioxide (TiO_2_) matrix [7]. Apparently, the incorporation of the Au NPs on various solid matrices (i.e., chitosan and TiO_2_) promotes the emergence of sensing ability, even at a considerably low RIS value. In fact, Au-TiO_2_ nanoplasmonic thin film, with sensitivity around ~33 nm/RIU, has been reported as a suitable sensing platform for biosensing detection [6,7]. In turn, Majdi et al. [31] reported colloidal chitosan-gold nanoparticles with an established RIS value of 60 nm RIU^−1^, determined by fitting of the LSPR band shift vs. the refractive index of several glycerol solutions. Indeed, several efforts should be made to enhance the sensing ability of the nanoplasmonic membrane developed in this work; however, the chitosan (0.5%)/Au-NPs plasmonic platform introduces a reasonable sensing potential to be applied as a transducer matrix in the development of a T-LSPR-based (bio)sensor.

### 3.4. T-LSPR Gas Sensing of Chitosan (0.5%)/Au-NPs Membrane

The gas sensing potential of the chitosan (0.5%)/Au-NPs plasmonic membrane was evaluated at room temperature by switching the gaseous atmosphere inside the HR-LSPR spectroscopy system between He and Ar, as described in Figure 2b. Their transmittance spectra were monitored in real time in each atmosphere for several cycles, with 60 spectra acquired in each cycle.

**Figure 6:**
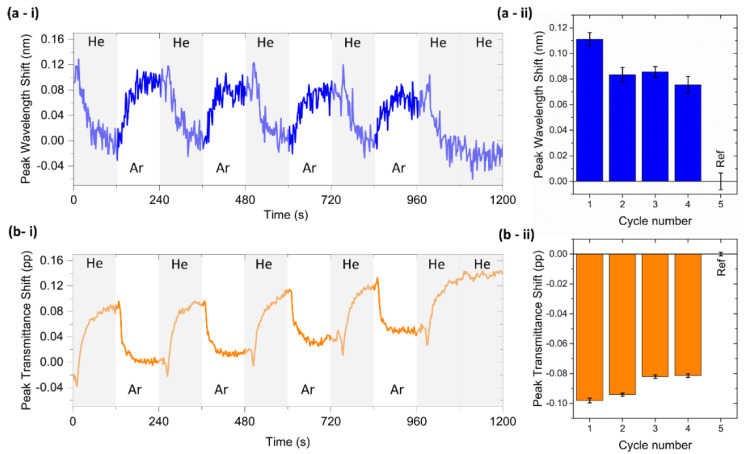
T-LSPR peak position monitoring of chitosan (0.5%)/Au-NPs for He and Ar cycles. Wavelength and transmittance coordinates shift of the T-LSPR peak over time ((**a-i**) and (**b-i**), respectively) and for 5 cycles ((**a-ii**) and (**b-ii**), respectively).

T-LSPR peak shifts were determined for both wavelength and transmittance coordinates (Figure 6). The cycles between each gas were perfectly perceptible over time Figure 6(a-i) in wavelength and Figure 6(b-i) in transmittance). The T-LSPR wavelength peak position underwent a redshift during Ar exposure and a blueshift after re-introducing the He gas, returning to its baseline value. Regarding the T-LSPR peak transmittance position, it shifted to lower transmittance values when Ar was introduced and to higher transmittance values when He was re-introduced. The reference cycle (the last cycle in the graphs) was made with two ‘atmospheres’ of He only, and no change was measured, thus confirming the different response of the chitosan (0.5%)/Au-NPs membrane to He and Ar atmospheres. The average wavelength (Figure 6(a-ii)) and transmittance shifts (Figure 6(b-ii)) were 0.089 ± 0.011 nm and −0.089 ± 0.007 pp, respectively. The possible reason for these T-LSPR band shifts is mainly related to the change in the RI of the bulk media from He to higher RI gaseous molecules (Ar). Hence, it was possible to estimate the RIS value of the sensor, being (1.5 ± 0.2) × 10^3^ nm/RIU and (−1.5 ± 0.1) × 10^3^ pp/RIU for wavelength and transmittance parameters, respectively. As different surrounding media (liquids and gases) are used to test the sensitivity of the sensor and assuming that their interaction with the nanoplasmonic membrane is thus different, their RIS values cannot be correlated. Chitosan membranes present a hydrophilicity nature with high permeability to water [32]. As a result, it preferentially permeates water rather than other molecules, such as sucrose, and thus the effective RI surrounding the Au NPs might be lower than expected. On the other hand, gas atoms such as Ar or He, due to their small size, can easily penetrate the membrane pores and increase the effective RI surrounding the NPs, leading to higher RIS values in gas sensing tests than in liquid tests. In the literature, no gas sensing tests have been performed to date with chitosan/Au-NPs membranes; however, their high RIS values are comparable to previously reported LSPR sensors [33,34].

Several cycles were performed, and membrane sensitivity was maintained; however other measurements are necessary to confirm long-term stability.

To evaluate the optical (T-LSPR) signal quality, the SNR of the chitosan (0.5%)/Au-NPs membrane was also calculated for both wavelength and transmittance parameters. Regarding the T-LSPR peak in the wavelength signal, the SNR was around 3.8; in turn, the T-LSPR peak in transmittance was about 15.5, a significantly higher value. Despite both being above the considerable acceptable value (SNR > 3) to estimate the detection limit [35], the transmittance parameter is more suitable for a good-quality measurement. Thus, considering an SNR = 3 and assuming that the SNR is scaled linearly with the RI difference, an LOD of the nanoplasmonic sensor of 1.2 × 10^−5^ RIU in transmittance is possible to be estimated.

## 4. Conclusions

A novel nanoplasmonic sensor based on Au NPs embedded in a chitosan membrane was developed, and its optical sensing ability was tested. Firstly, several chitosan/Au-NPs membranes were produced by the spin-coating technique, using four different polymer concentrations. The lowest chitosan concentration (chitosan (0.5%)/Au-NPs membrane) presented the most suitable optical (T-LSPR) response, namely a narrower and well-defined T-LSPR band. Hereupon, the sensing ability of the chitosan (0.5%)/Au-NPs membrane was investigated through two different sensitivity tests, inducing the medium’s refractive index changes, either in a liquid or gas media. Both preliminary sensing analyses revealed a potentially versatile nanoplasmonic sensor, based on the chitosan (0.5%)/Au-NPs membrane, since it was capable of transducing different chemical environments/signals to an output optical (plasmonic) signal. Hence, this nanoplasmonic membrane is expected to be a suitable transducer platform for different relevant LSPR-based applications, not only for chemo-sensing, but also for physical sensors as flexible (strain) sensors or others. However, optimization of the nanoplasmonic membrane production (e.g., the influence of spin-coating speed/rotation, time, temperature, membrane thickness, and Au concentration) and post-production treatment can be beneficial to improve its sensing performance.

## Data Availability

Not applicable.

## References

[1] Rodrigues M.S., Borges J., Lopes C., Pereira R.M.S., Vasilevskiy M.I., Vaz F. (2021). Gas Sensors Based on Localized Surface Plasmon Resonances: Synthesis of Oxide Films with Embedded Metal Nanoparticles, Theory and Simulation, and Sensitivity Enhancement Strategies. Appl. Sci..

[2] Ha M., Kim J.H., You M., Li Q., Fan C., Nam J.M. (2019). Multicomponent Plasmonic Nanoparticles: From Heterostructured Nanoparticles to Colloidal Composite Nanostructures. Chem. Rev..

[3] Liu J., He H., Xiao D., Yin S., Ji W., Jiang S., Luo D., Wang B., Liu Y. (2018). Recent Advances of Plasmonic Nanoparticles and Their Applications. Materials.

[4] Proença M., Rodrigues M.S., Borges J., Vaz F. (2020). Optimization of Au:CuO Nanocomposite Thin Films for Gas Sensing with High-Resolution Localized Surface Plasmon Resonance Spectroscopy. Anal. Chem..

[5] Meira D.I., Domingues R.P., Rodrigues M.S., Alves E., Barradas N.P., Borges J., Vaz F. (2020). Thin Films of Au-Al_2_O_3_ for Plasmonic Sensing. Appl. Surf. Sci..

[6] Barbosa A.I., Borges J., Meira D.I., Costa D., Rodrigues M.S., Rebelo R., Correlo V.M., Vaz F., Reis R.L. (2019). Development of Label-Free Plasmonic Au-TiO_2_ Thin Film Immunosensor Devices. Mater. Sci. Eng. C.

[7] Pereira-Silva P., Meira D.I., Costa-Barbosa A., Costa D., Rodrigues M.S., Borges J., Machado A.V., Cavaleiro A., Sampaio P., Vaz F. (2022). Immobilization of Streptavidin on a Plasmonic Au-TiO_2_ Thin Film towards an LSPR Biosensing Platform. Nanomater.

[8] Sun L.L., Leo Y.S., Zhou X., Ng W., Wong T.I., Deng J. (2020). Localized Surface Plasmon Resonance Based Point-of-Care System for Sepsis Diagnosis. Mater. Sci. Energy Technol..

[9] Zhang P., Chen Y.-P., Wang W., Shen Y., Guo J.-S. (2016). Surface Plasmon Resonance for Water Pollutant Detection and Water Process Analysis. TrAC Trends Anal. Chem..

[10] Li D., Zhang Y., Guo Q., Sun X., Zhang H., Wang S., Birech Z., Hu J. (2020). An Efficient LSPR Method to Quantitatively Detect Dimethoate: Development, Characterization and Evaluation. PLoS ONE.

[11] Wang W., You Y., Gunasekaran S. (2021). LSPR-Based Colorimetric Biosensing for Food Quality and Safety. Compr. Rev. Food Sci. Food Saf..

[12] Unser S., Bruzas I., He J., Sagle L. (2015). Localized Surface Plasmon Resonance Biosensing: Current Challenges and Approaches. Sensors.

[13] Kasani S., Curtin K., Wu N. (2019). A Review of 2D and 3D Plasmonic Nanostructure Array Patterns: Fabrication, Light Management and Sensing Applications. Nanophotonics.

[14] Proença M., Borges J., Rodrigues M.S., Meira D.I., Sampaio P., Dias J.P., Pedrosa P., Martin N., Bundaleski N., Teodoro O.M.N.D. (2019). Nanocomposite Thin Films Based on Au-Ag Nanoparticles Embedded in a CuO Matrix for Localized Surface Plasmon Resonance Sensing. Appl. Surf. Sci..

[15] Rodrigues M.S., Borges J., Vaz F. (2022). Plasmonic Strain Sensors Based on Au-TiO_2_ Thin Films on Flexible Substrates. Sensors.

[16] Rodrigues M.S., Borges J., Vaz F. (2020). Enhancing the Sensitivity of Nanoplasmonic Thin Films for Ethanol Vapor Detection. Materials.

[17] Xiang G., Zhang N., Zhou X. (2010). Localized Surface Plasmon Resonance Biosensing with Large Area of Gold Nanoholes Fabricated by Nanosphere Lithography. Nanoscale Res. Lett..

[18] Miranda B., Rea I., Dardano P., De Stefano L., Forestiere C. (2021). Recent Advances in the Fabrication and Functionalization of Flexible Optical Biosensors: Toward Smart Life-Sciences Applications. Biosensors.

[19] Karakouz T., Vaskevich A., Rubinstein I. (2008). Polymer-Coated Gold Island Films as Localized Plasmon Transducers for Gas Sensing. J. Phys. Chem..

[20] Hanif M., Juluri R.R., Fojan P., Popok V. (2016). Polymer Films with Size-Selected Silver Nanoparticles as Plasmon Resonance-Based Transducers for Protein Sensing. Biointerface Res. Appl. Chem..

[21] Bhattarai J.K., Uddin Maruf M.H., Stine K.J. (2020). Plasmonic-Active Nanostructured Thin Films. Processes.

[22] Rivero P.J., Hernaez M., Goicoechea J., Matias I.R., Arregui F.J. Optical Fiber Refractometers Based on Localized Surface Plasmon Resonance (LSPR) and Lossy Mode Resonance (LMR). Proceedings of the 23rd International Conference on Optical Fibre Sensors.

[23] Rivero P.J., Goicoechea J., Arregui F.J. (2018). Optical Fiber Sensors Based on Polymeric Sensitive Coatings. Polymers.

[24] Hahn T., Tafi E., Paul A., Salvia R., Falabella P., Zibek S. (2020). Current State of Chitin Purification and Chitosan Production from Insects. J. Chem. Technol. Biotechnol..

[25] Casadidio C., Peregrina D.V., Gigliobianco M.R., Deng S., Censi R., Di Martino P. (2019). Chitin and Chitosans: Characteristics, Eco-Friendly Processes, and Applications in Cosmetic Science. Mar. Drugs.

[26] Rodrigues M.S., Pereira R.M.S., Vasilevskiy M.I., Borges J., Vaz F. (2020). NANOPTICS: In-Depth Analysis of NANomaterials for OPTICal Localized Surface Plasmon Resonance Sensing. SoftwareX.

[27] Kedem O., Vaskevich A., Rubinstein I. (2014). Critical Issues in Localized Plasmon Sensing. J. Phys. Chem. C.

[28] Alex S., Tiwari A. (2015). Functionalized Gold Nanoparticles: Synthesis, Properties and Applications-A Review. J. Nanosci. Nanotechnol..

[29] Lopatina L.I., Tsarkova L.A. (2019). PH-Triggered Aggregation Behavior of Hybrid Chitosan Assemblies with Controlled Density Distribution of Gold Nanoparticles. Colloid Polym. Sci..

[30] Chen R., Sun Y., Huo B., Zhao X., Huang H., Li S., Bai J., Liang J., Gao Z. (2021). A Copper Monosulfide-Nanoparticle-Based Fluorescent Probe for the Sensitive and Specific Detection of Ochratoxin A. Talanta.

[31] Majdi H., Salehi R., Pourhassan-Moghaddam M., Mahmoodi S., Poursalehi Z., Vasilescu S. (2019). Antibody Conjugated Green Synthesized Chitosan-Gold Nanoparticles for Optical Biosensing. Colloids Interface Sci. Commun..

[32] Alshahrani A.A., Alsuhybani M., Algamdi M.S., Alquppani D., Mashhour I., Alshammari M.S., Alsohaimi I.H., Alraddadi T.S. (2021). Evaluating the Performance of Chitosan and Chitosan-Palm Membrane for Water Treatment: Preparation, Characterization and Purification Study. J. Taibah Univ. Sci..

[33] Proença M., Rodrigues M.S., Meira D.I., Castro M.C.R., Rodrigues P.V., Machado A.V., Alves E., Barradas N.P., Borges J., Vaz F. (2022). Optimization of Au:CuO Thin Films by Plasma Surface Modification for High-Resolution LSPR Gas Sensing at Room Temperature. Sensors.

[34] Jeong H.-H., Mark A.G., Alarcón-Correa M., Kim I., Oswald P., Lee T.-C., Fischer P. (2016). Dispersion and Shape Engineered Plasmonic Nanosensors. Nat. Commun..

[35] Djurić Z., Jokić I., Milovanović G. Signal-to-Noise Ratio in Adsorption-Based Microfluidic Bio/Chemical Sensors. Proceedings of the 30th Anniversary Eurosensors Conference–Eurosensors 2016.

